# The Cardiovascular Burden of Diabetes: Risk Factors, Clinical Phenotypes, and Personalized Cardiometabolic Management

**DOI:** 10.3390/jcm15062358

**Published:** 2026-03-19

**Authors:** Giuliano Cassataro, Giulio Geraci, Maria Ausilia Giusti, Carlo Maida, Viviana Maggio, Manfredi Rizzo, Alessandro Mattina

**Affiliations:** 1 Internal Medicine and Pulmonology Unit, Fondazione Istituto “G. Giglio”, 90015 Cefalù, Italy; 2 Department of Medicine and Surgery, “Kore” University of Enna, 94100 Enna, Italy; 3 Internal Medicine Unit, Hospital Umberto I, 94100 Enna, Italy; 4 UPMC Italy (University of Pittsburgh Medical Center), 90127 Palermo, Italy; 5 Diabetes Service, IRCCS ISMETT, 90127 Palermo, Italy; 6 Department of Internal Medicine, S. Elia Hospital, 93100 Caltanissetta, Italy; 7 Department of Health Promotion, Mother and Childcare, Internal Medicine and Medical Specialties, School of Medicine, University of Palermo, 90127 Palermo, Italy; 8 Internal Medicine Department, College of Medical Sciences, Ras Al Khaimah Medical and Health Sciences University, Ras Al Khaimah 11172, United Arab Emirates

**Keywords:** type 2 diabetes mellitus, cardiometabolic risk, atherosclerotic cardiovascular disease, heart failure, endothelial and microvascular dysfunction

## Abstract

Type 2 diabetes (T2D) exhibits substantial phenotypic heterogeneity, resulting in diverse cardiovascular (CV) outcomes driven by multiple pathophysiological mechanisms beyond hyperglycemia alone. T2D should be recognized as a systemic cardiometabolic condition in which insulin resistance, chronic inflammation, oxidative stress, and endothelial and microvascular dysfunction promote a broad spectrum of cardiovascular diseases. The traditional “one-size-fits-all” approach to cardiovascular risk management has been proven insufficient, as individuals with T2D display marked variability in clinical presentation, disease trajectory, treatment response, and cardiovascular phenotype. In this context, personalized medicine strategies integrating clinical phenotyping, individualized risk stratification, and tailored therapeutic interventions offer the potential to optimize cardiometabolic outcomes while minimizing treatment burden and adverse effects. This narrative review examines the rationale and current evidence supporting personalized cardiovascular risk management in T2D. We discuss the heterogeneity of diabetes-related CV phenotypes, encompassing both atherosclerotic and non-atherosclerotic complications. We further examine the major cardiometabolic risk factors closely linked to diabetes, including dyslipidemia, hypertension, obesity, chronic kidney disease, and metabolic liver disease, which act synergistically to accelerate vascular damage and end-organ injury, and are essential for defining personalized prognostic and therapeutic programs. Finally, we present structured approaches to cardiovascular assessment and highlight contemporary management strategies that prioritize integrated, phenotype-driven risk reduction using cardioprotective glucose-lowering therapies together with optimized lipid-lowering, antihypertensive, antithrombotic, and weight-modifying interventions. The transition from population-based guidelines to individualized, patient-centered care represents a paradigm shift in diabetes management, with the potential to substantially reduce the excess CV burden associated with this condition.

## 1. Introduction

The management of non-communicable diseases, particularly type 2 diabetes (T2D) and its cardiovascular (CV) complications, stands at a critical juncture. Traditional population-based approaches have yielded important advances, yet substantial residual risk persists even with optimal implementation of standard therapies. Emerging evidence reveals that diabetes is not a single entity but rather an umbrella term encompassing multiple pathophysiological subtypes with distinct genetic, metabolic, and clinical profiles. This heterogeneity extends to CV manifestations, treatment responses, and long-term outcomes, underscoring the urgent need for personalized medicine approaches that move beyond ‘one-size-fits-all’ protocols to deliver precision interventions tailored to individual patient characteristics, phenotypes, and risk profiles.

Around 536 million adults are currently living with diabetes, marking a significant increase from approximately 200 million in 1990. Projections suggest this number will rise further to 643 million by 2030 and 783 million by 2045. This rise is particularly pronounced in low- and middle-income countries [1].

T2D is frequently associated with obesity, sedentary lifestyles, and genetic factors. Its long-term implications are serious and include an increased risk of cardiovascular diseases (CVDs), kidney failure, neuropathy, retinopathy, and other multi-organ complications [2,3]. The heightened CVD risk in diabetes consists of a spectrum of conditions, including coronary artery disease (CAD), atrial fibrillation (AF), stroke, and heart failure (HF). CV risk is markedly elevated in individuals with T2D, even in the absence of established atherosclerotic disease, and remains the leading cause of morbidity and mortality in this population [2,4].

Documented evidence underscores that the relative risk of CVDs is approximately twofold higher in individuals with diabetes compared to those without [3]. In studies that included gender-specific prevalence rates, males showed higher prevalence rates for most CV outcomes compared to females but women with diabetes have a greater relative risk of CAD and all-cause mortality [5]. For people with diabetes, the overall prevalence of CVD was found to be 32.2%, with CAD and HF being the most prevalent comorbidities, recorded at 21.2% and 14.9%, respectively [6]. The global burden of CVDs and their risk factors have been closely examined, revealing substantial disparities across different demographics and regions driven by socioeconomic factors, healthcare access, and cultural dietary practices [7]. The relationship between diabetes and CVD is particularly pronounced in older populations, where the prevalence of diagnosed diabetes escalates with age. Globally, the prevalence of diabetes among individuals aged 65 and older is estimated to exceed 25%, with significant variations between high-income and low-income countries [8]. Additionally, disparities in diabetes prevalence exist among ethnic groups, with genetic predispositions and lifestyle factors contributing to these differences [1]. To address the global impact of diabetes on CV health, international efforts must focus on equitable healthcare delivery and culturally sensitive interventions. Taken together, these data indicate that diabetes and CVDs are intricately linked through a complex network of shared risk factors and pathophysiological mechanisms.

**Figure 1:**
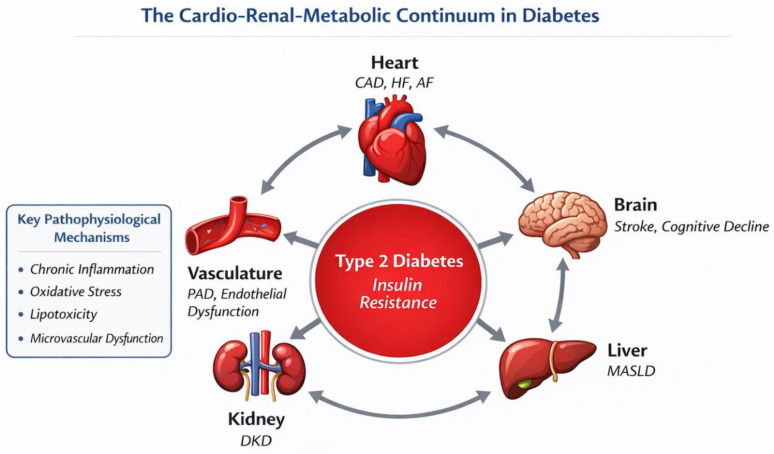
The cardio–renal–metabolic continuum in type 2 diabetes. Type 2 diabetes, driven by systemic insulin resistance, acts as a central pathological hub linking cardiovascular, renal, hepatic, cerebral, and vascular dysfunction. Shared mechanisms—including chronic low-grade inflammation, oxidative stress, lipotoxicity, and microvascular dysfunction—contribute to the development and progression of coronary artery disease, heart failure, atrial fibrillation, stroke and cognitive decline, diabetic kidney disease, metabolic-dysfunction-associated steatotic liver disease, and peripheral arterial disease. The bidirectional interactions among organs highlight the systemic nature of diabetes and support the need for integrated, multidisciplinary cardiovascular risk management. CAD: coronary artery disease, HF: heart failure, AF: atrial fibrillation, MASLD: metabolic-dysfunction-associated steatotic liver disease, DKD: diabetic kidney disease, PAD: peripheral arterial disease.

Rather than acting as an isolated metabolic disorder, T2D represents a systemic condition in which insulin resistance and common biological pathways drive multisystem organ damage, giving rise to a broad spectrum of CV and extracardiac manifestations (Figure 1). This article therefore aims to summarize current evidence on epidemiology, underlying mechanisms, clinical manifestations, diagnostic evaluation, and management strategies of diabetes-associated CVDs.

This narrative review was based on a structured search of PubMed/MEDLINE and Scopus for articles published between 2015 and January 2026. The search included combinations of keywords such as type 2 diabetes, cardiovascular disease, cardiorenal risk, heart failure, atherosclerosis, precision medicine, and phenotype-driven management. Priority was given to randomized controlled trials (RCTs), large observational studies, meta-analyses, and major international guidelines (ESC, ADA, EASD). Additional references were identified through manual screening of relevant bibliographies. Studies were selected based on their relevance to cardiovascular phenotypes, risk stratification, and cardiometabolic management in T2D.

## 2. Shared Pathophysiological Mechanisms Linking Diabetes and Cardiovascular Disease

Diabetes-associated CVDs are driven by a set of shared and interrelated pathophysiological mechanisms, a pro-atherogenic and pro-inflammatory milieu sustained by chronic hyperglycemia, dyslipidemia, and ectopic lipid accumulation.

Chronic low-grade inflammation represents a key unifying mechanism. Hyperglycemia and excess free fatty acids activate inflammatory signaling pathways, leading to increased production of cytokines such as interleukin-6 (IL-6) and tumor necrosis factor-α (TNF-α), which contribute to endothelial dysfunction, vascular remodeling, and myocardial fibrosis [9].

Oxidative stress and the formation of advanced glycation end-products (AGEs) further amplify vascular and myocardial injury. Persistent hyperglycemia favors the accumulation of AGEs within the vessel wall and myocardium, where they impair nitric oxide bioavailability, increase arterial stiffness, and promote microvascular dysfunction. AGEs exert potent biological actions through binding to their specific receptors (RAGE), which activates intracellular pro-inflammatory and pro-thrombotic signaling pathways. This AGE–RAGE axis sustains endothelial activation, enhances vascular permeability, and increases thrombogenic potential, thereby linking metabolic dysregulation to progressive vascular injury [10].

Taken together, these shared mechanisms provide a common biological substrate underlying the heterogeneous CV phenotypes observed in diabetes and explain the persistence of CV risk despite adequate glycemic control alone [11]. Microvascular dysfunction and myocardial fibrosis preferentially contribute to HFpEF and coronary microvascular disease, whereas systemic inflammation and atherogenic dyslipidemia accelerate atherosclerotic phenotypes. This biological heterogeneity provides the rationale for a phenotype-oriented clinical approach.

## 3. Clinical Manifestations of Diabetes-Associated Cardiovascular Disease

CVDs represent the leading cause of morbidity and mortality among individuals with diabetes. Despite optimal glycemic control, substantial residual CV risk persists, reflecting the combined impact of hypertension, dyslipidemia, adiposity, and physical inactivity [12]. The clustering of cardiovascular complications in T2D gives rise to distinct clinical phenotypes, including atherosclerotic, heart failure/microvascular, and cardiometabolic patterns.

Coronary artery disease (CAD) is the most prevalent CV manifestation in diabetic patients with progressive atherosclerosis affecting both epicardial coronary arteries and the coronary microcirculation [13]. In this setting, CAD may assume a distinct and more aggressive phenotype, marked by diffuse and progressive coronary involvement and associated with a high burden of subclinical disease [14,15].

Beyond epicardial disease, diabetes mellitus is strongly associated with coronary microvascular dysfunction, as reflected by impaired coronary flow reserve (CFR), even in the absence of obstructive coronary artery disease [16]. Reduced CFR, documented by Doppler-based studies in diabetic populations, represents a powerful predictor of myocardial ischemia, adverse CV outcomes and heart failure (HF).

HF, in particular, is a common and clinically relevant complication of T2D, encompassing a broad spectrum of phenotypes defined by left ventricular ejection fraction. Diabetes affects up to 40% of patients with chronic or acute HF and independently increases the risk of HF by approximately 2.5-fold, reflecting the combined impact of myocardial ischemia, metabolic derangements, and direct myocardial injury [17]. Diabetic cardiomyopathy represents a distinct pathway to heart failure, characterized by left ventricular hypertrophy, diastolic dysfunction, and myocardial fibrosis, and occurring independently of overt ischemic heart disease [2]. These mechanisms preferentially promote the development of heart failure with preserved ejection fraction (HFpEF), which represents the predominant HF phenotype in diabetes. Microvascular rarefaction and impaired myocardial perfusion contribute to left ventricular diastolic dysfunction even in the absence of overt coronary artery disease. Consistent with this concept, data from the Framingham Heart Study demonstrate that diabetes markedly increases the risk of HFpEF independently of coronary artery disease and hypertension, underscoring the central role of microvascular injury in diabetes-related heart failure.

Peripheral arterial disease (PAD) represents another major CV manifestation of diabetes and serves as a sentinel marker of systemic atherosclerosis and heightened CV risk. In individuals with diabetes, PAD is typically more diffuse and distal, reflecting the systemic inflammatory, endothelial, and microvascular alterations that characterize diabetes-related vascular disease [18]. Diabetes markedly increases the risk of PAD, acting as an independent determinant of both symptomatic and asymptomatic disease [19]. Ankle–brachial index (ABI) measurement is the tool for diagnosing PAD, with reduced ABI values (<0.9) identifying diabetic patients at higher risk of major CV events, including myocardial infarction and stroke [20]. Management of PAD in diabetes requires a comprehensive approach targeting global CV risk. While intensive glycemic control may reduce overall CV events, its effect on PAD progression remains uncertain [21]. Optimal management therefore relies on aggressive modification of concomitant risk factors—particularly hypertension and smoking—together with structured lifestyle interventions, including supervised exercise, to improve functional status and reduce long-term CV risk.

Finally, cerebrovascular disease represents one of the most serious and potentially debilitating complications of diabetes, encompassing transient ischemic attacks (TIA) and both ischemic and hemorrhagic strokes. Large population studies and meta-analyses consistently show that individuals with T2D have a two- to three-fold higher risk of stroke compared with those without diabetes, highlighting the substantial cerebrovascular burden in this population [21]. Diabetes contributes to cerebrovascular injury through a combination of endothelial dysfunction, accelerated atherosclerosis, and microvascular damage affecting cerebral circulation [22]. Although improved glycemic control clearly reduces microvascular complications, its effect on major cerebrovascular events appears limited, highlighting the persistence of residual risk and the importance of multifactorial CV prevention beyond glucose lowering alone [23]. In contrast, effective blood pressure control has been shown to significantly reduce stroke incidence in patients with diabetes, reinforcing hypertension as a key modifiable determinant of cerebrovascular risk [24]. Diabetes is also associated with multiple ischemic stroke subtypes. Large-artery atherosclerosis represents a frequent mechanism; additionally, diabetes increases the risk of atrial fibrillation, thereby contributing to cardioembolic stroke risk [25]. Small-vessel disease leading to lacunar infarction is particularly common in diabetic patients, reflecting diabetes-related cerebral microangiopathy and conferring an increased risk of recurrent events [26]. In addition, diabetes may contribute to ischemic stroke through less common mechanisms, including hypercoagulability, and through overlapping cardiometabolic risk factors that may complicate etiological classification in a subset of patients.

## 4. The Linkage Between Diabetes and CV Risk Factors

The excess CV risk associated with diabetes arises from the clustering and interaction of multiple traditional and diabetes-specific risk factors, which act in synergy to accelerate vascular damage and end-organ complications.

### 4.1. DM and Dyslipidemia

A pivotal component of dyslipidemia in diabetic patients is insulin resistance, which stimulates an increased release of free fatty acids from adipose tissue. This leads to enhanced triglyceride synthesis within the liver, resulting in elevated levels of triglyceride-rich very low-density lipoprotein (VLDL) and low-density lipoprotein (LDL) particles, both associated with increased atherogenic potential. Furthermore, altered lipoprotein metabolism, particularly the proliferation of small dense LDL particles, heightens CV risk due to their elevated propensity to accelerate atherosclerosis [27,28]. In addition to these lipid abnormalities, diabetic patients often exhibit reduced levels of high-density lipoprotein (HDL) cholesterol. Lower HDL levels impair this protective mechanism, further contributing to CV risk. Recent evidence suggests that low HDL cholesterol is an independent factor not only for CVDs but also for the development of diabetes itself [29].

Elevated triglyceride levels are another common feature in diabetic dyslipidemia. High triglycerides are associated with an increased risk of CV events, as they contribute to the formation of small dense LDL particles and may promote endothelial dysfunction [30,31]. An important role in defining residual CV risk, is played by lipoprotein (a) [Lp(a)]: its measurement is recommended to improve risk stratification in patients at intermediate or high risk, particularly in the presence of aortic stenosis or elevated CV risk [32].

### 4.2. DM and Hypertension

The association between diabetes and hypertension is of major clinical relevance, as approximately 50–80% of individuals with T2D are affected by hypertension. Their coexistence markedly worsens prognosis, elevating the risk of both microvascular and macrovascular complications, including myocardial infarction and stroke [33]. In T2D patients, hypertension often precedes the onset of diabetes, whereas in type 1 diabetes (T1D), it frequently develops as a consequence of diabetic nephropathy. Multiple pathophysiological mechanisms underline this association. Persistent hyperglycemia and insulin resistance promote renal sodium retention, activation of the sympathetic nervous system, endothelial dysfunction, and chronic low-grade inflammation, collectively leading to increased arterial stiffness and elevated blood pressure levels [33]. Clinical management of patients with coexisting diabetes and hypertension requires a multifaceted approach, combining lifestyle interventions, pharmacological treatment, and close monitoring. Angiotensin-converting enzyme (ACE) inhibitors and angiotensin II receptor blockers (ARBs) are preferred antihypertensive agents due to their nephroprotective properties and CV benefits, while tight glycemic control and dietary sodium restriction remain essential components of comprehensive risk reduction strategies [34].

### 4.3. DM and Obesity

Obesity acts as a major modifier of cardiometabolic risk by amplifying the shared inflammatory and insulin-resistant mechanisms. Excess visceral adiposity is associated with adipokine imbalance, including leptin resistance and reduced adiponectin levels, leading to impaired insulin signaling, dysregulated energy homeostasis, and adverse vascular effects [35,36]. Leptin resistance blunts its physiological anorexigenic and metabolic actions while sustaining pro-inflammatory signaling, whereas reduced adiponectin removes an important anti-inflammatory and insulin-sensitizing influence [37]. In parallel, obesity-related low-grade inflammation, characterized by macrophage infiltration of adipose tissue, further exacerbates insulin resistance and vascular dysfunction, thereby increasing atherosclerotic risk [38].

The management of obesity in diabetic patients is a crucial component of reducing CV risk [39]. Lifestyle interventions, including dietary modifications and increased physical activity, have been shown to improve insulin sensitivity and endothelial function [40]. Pharmacological treatments targeting obesity, such as glucagon-like peptide-1 receptor agonists (GLP-1 RAs) and sodium–glucose cotransporter-2 inhibitors (SGLT2i), have demonstrated beneficial effects on body weight reduction, glycemic control, and CV protection. While SGLT2i provide modest but consistent benefits on body weight together with robust cardiorenal protection, GLP-1 RAs achieve greater weight reduction and broader cardiometabolic improvements. More recently, dual incretin agonists have demonstrated the most pronounced effects on body weight and glycemic control. The comparative mechanisms of action and clinical outcome evidence supporting these therapies are discussed in detail in the pharmacological management section.

### 4.4. DM and Atrial Fibrillation

Diabetes mellitus (DM) is a risk factor for atrial fibrillation (AF). The association between diabetes and AF reflects a complex interplay of structural, electrical, and autonomic atrial remodeling. Insulin resistance plays a central role by promoting sympathetic overactivation and cardiac ion channel dysregulation, thereby increasing atrial electrical instability and susceptibility to AF [41]. In parallel, diabetes-related myocardial changes—including fibrosis, diastolic dysfunction, oxidative stress, and advanced glycation end-product accumulation (AGEs)—contribute to atrial enlargement, conduction abnormalities and pro-arrhythmogenic substrate [42]. Altered autonomic balance, characterized by increased sympathetic tone and reduced parasympathetic modulation, further shorten atrial refractoriness and facilitates ectopic activity [43]. The coexistence of diabetes and AF markedly increases thromboembolic risk, particularly stroke, owing to enhanced platelet activity, hypercoagulability, and endothelial dysfunction [44,45]. Management of AF in patients with diabetes therefore requires an integrated approach combining rhythm and rate control, weight reduction, and comprehensive CV prevention.

### 4.5. DM and Renal Disease

Chronic hyperglycemia leads to glomerular hyperfiltration, podocyte injury, and the accumulation of extracellular matrix proteins, ultimately resulting in glomerulosclerosis and a decline in kidney function [46]. The activation of the renin–angiotensin–aldosterone system (RAAS) in diabetes further exacerbates renal damage by increasing intraglomerular pressure and promoting inflammation [47]. Albuminuria, a hallmark of diabetic nephropathy, is strongly associated with an elevated risk of CVDs, underscoring the interplay between diabetes, renal disease, and adverse CV outcomes [48]. This cardio–renal interplay is particularly relevant in patients progressing to end-stage kidney disease and kidney transplantation, where diabetes remains a major driver of post-transplant CV morbidity and mortality [49]. Robust evidence supports the use of SGLT2i as foundational therapy in diabetic kidney disease, conferring kidney protection and CV benefit.

In DAPA-CKD, dapagliflozin reduced the risk of a composite renal endpoint (sustained eGFR decline ≥50%, end-stage kidney disease, or death from renal or CV causes) in patients with CKD, including those with T2D [50]. Similarly, EMPA-KIDNEY demonstrated that empagliflozin lowered the risk of kidney disease progression or CV death across a broad CKD population, reinforcing a class effect on cardiorenal outcomes [51]. In addition, targeting mineralocorticoid-receptor-mediated inflammation and fibrosis with the non-steroidal mineralocorticoid receptor antagonist finerenone provides incremental benefit on top of optimized RAAS inhibition. In FIDELIO-DKD, finerenone reduced CKD progression and CV events in patients with CKD and T2D, while FIGARO-DKD showed a significant reduction in CV outcomes, particularly heart-failure-related events, in a complementary CKD phenotype [52,53]. Collectively, these data support a contemporary multi-pathway approach (RAAS blockade + SGLT2i ± finerenone) to slow diabetic kidney disease progression and reduce residual CV risk.

### 4.6. DM and MASLD

Metabolic-Dysfunction-Associated Steatotic Liver Disease (MASLD) is highly prevalent in patients with diabetes: [54]. MASLD shares with diabetes and obesity a state of chronic low-grade inflammation and oxidative stress, as described above, which exacerbate endothelial dysfunction and accelerate atherosclerosis [55,56]. As a result, MASLD significantly amplifies CV risk in diabetes; indeed, CV disease is the leading cause of death in MASLD, and the risk of mortality is even higher in those with both MASLD and T2D [54]. Recognizing this dual hazard, recent clinical guidelines now advocate screening for MASLD in patients with diabetes—particularly to detect advanced fibrosis—even if liver enzymes are normal [57]. This typically involves non-invasive fibrosis assessment (e.g., Fibrosis-4 score, elastography) to identify patients who may benefit from early interventions or specialist referral. Beyond lifestyle modification and glucose-lowering therapies, novel pharmacological agents specifically targeting MASH pathophysiology have recently shown promising results with potential implications for CV risk reduction. In the MAESTRO-NASH trial, resmetirom led to a significantly higher rate of MASH resolution without worsening of fibrosis and improvement in fibrosis stage compared with placebo, alongside favorable effects on lipid parameters, including reductions in LDL cholesterol and atherogenic lipoproteins [58]. These pleiotropic metabolic effects suggest a potential role for resmetirom in mitigating both hepatic and CV risk in patients with MASLD and T2D. In parallel, incretin-based therapies have shown increasing relevance in the treatment of MASLD/MASH. In the ESSENCE trial, high-dose semaglutide 2.4 mg significantly improved histological features of MASH, including resolution of steatohepatitis, while inducing substantial and sustained weight loss and improving cardiometabolic risk factors [59]. Given the established CV benefits of GLP-1 RAs in patients with T2D, these findings reinforce the concept that therapies addressing metabolic dysfunction, hepatic disease, and CV risk simultaneously may represent a paradigm shift in the integrated management of diabetes-associated MASLD.

These intricate mechanisms underscore the need for a comprehensive approach to managing CV risk factors in diabetic populations. A structured overview of the major CV risk factors in diabetes is summarized in Table 1.

## 5. Structured Cardiovascular Workup in Diabetes

A structured CV evaluation is essential in patients with diabetes to ensure personalized care tailored to disease complexity and comorbidities, which should be approached within a cardiorenal metabolic framework [62]. According to the 2023 ESC Guidelines for the management of CVDs in patients with diabetes, all patients should undergo systematic assessment for CVDs, including medical history and evaluation of symptoms suggestive of CV involvement [61]. First-level CV evaluation includes an electrocardiogram (ECG); in this context, screening for AF by pulse taking or ECG is recommended in patients ≥65 years of age [63]. Transthoracic echocardiography complements ECG by assessing cardiac structure and function, including systolic and diastolic performance, valvular disease, and pulmonary pressures and plays a pivotal role in identifying early manifestations of heart failure, particularly HFpEF [64]. In addition, guideline-directed screening for heart failure through clinical assessment is recommended at each visit, with measurement of BNP or NT-proBNP when heart failure is suspected [65].

Carotid ultrasound may be considered in selected high-risk patients to refine CV risk stratification. In patients with diabetes, the presence of carotid plaque represents an early marker of target organ damage, even in the absence of overt CVD [66]. Second-level investigations are reserved for patients requiring further diagnostic strategies according to individual risk profiles [67]. Commonly used second-line tests include exercise ECG testing and stress echocardiography. To further improve CV risk stratification in T2D, diabetes-specific risk prediction tools have been developed. SCORE2-Diabetes estimates 10-year risk of myocardial infarction and stroke by integrating traditional CV risk factors with diabetes-specific variables [68]. Additionally, the DIAL2 model provides estimates of lifetime CV disease-free life expectancy in individuals with T2D without established CVD, particularly in European low- and moderate-risk regions, supporting shared decision-making in accordance with the 2021 ESC Guidelines on CVD prevention [69,70].

**Figure 2:**
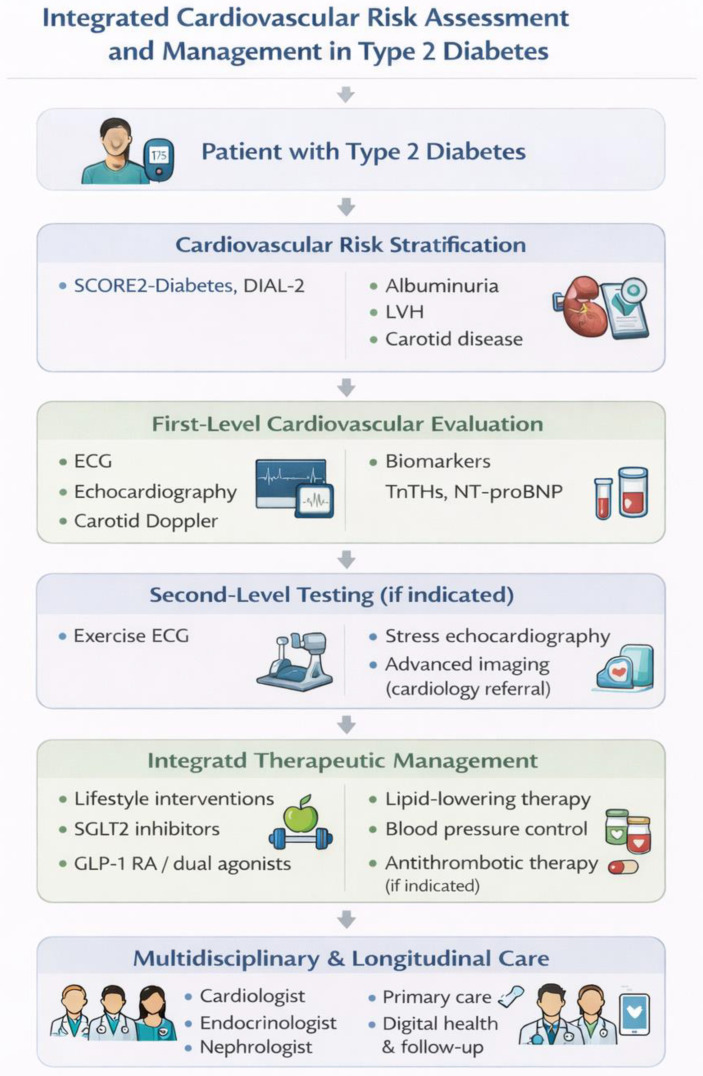
Integrated cardiovascular risk assessment and management in type 2 diabetes. A stepwise clinical approach combining diabetes-specific risk stratification tools, first- and second-level cardiovascular investigations, and comprehensive therapeutic strategies. The model emphasizes the central role of lifestyle modification, cardioprotective glucose-lowering therapies, and optimal control of blood pressure, lipids, and thrombosis risk, within a multidisciplinary and longitudinal care framework. ECG: electrocardiogram, hs-TnT: high-sensitivity cardiac troponin T, NT-proBNP: N-terminal pro–B-type natriuretic peptide, SGLT2: sodium–glucose cotransporter 2, GLP-1 RA: glucagon-like peptide-1 receptor agonist.

Overall, a comprehensive and guideline-driven CV workup is crucial for early detection and optimal management of CVD in diabetes. Such structured and multidisciplinary CV assessment is especially warranted in complex populations, including solid organ transplant recipients with diabetes, who often exhibit accelerated CV risk trajectories. An integrated and stepwise approach to CV risk assessment and management in patients with T2D is summarized in Figure 2.

## 6. Clinical Management and Therapeutic Approaches

The management of CVD starts with lifestyle interventions and pharmacotherapy aimed at controlling lipids, hypertension, hyperglycemia, and obesity. Additionally, antiplatelet and anticoagulant therapies are implemented as appropriate for individual patient needs. A multi-faceted strategy is essential to address the dual burden of diabetes and CVDs.

### 6.1. Lifestyle Modifications According to Cultural and Regional Disparities

The relationship between diabetes and CVDs is shaped not only by individual risk factors but also by regional, socioeconomic and cultural determinants that influence dietary patterns, physical activity and access to preventive care. Global comparative risk assessments consistently demonstrate substantial geographic heterogeneity in cardiometabolic risk with suboptimal diet representing one of the leading contributors to the global burden of non-communicable diseases [71]. Similarly, Global Burden of Disease analyses confirm that CVD and key drivers, including diabetes, obesity, and hypertension, remain major contributors to mortality and disability across regions, with divergent trends between high-income and low- and middle-income settings [7].

Importantly, beyond exposure to traditional risk factors, disparities in healthcare access and quality critically influence cardiometabolic outcomes. In many low- and middle-income settings, significant gaps persist along the diabetes and hypertension care cascades—from early detection to treatment initiation and adequate risk-factor control—potentially amplifying the burden of cardiovascular complications [72]. These structural determinants highlight that optimal cardiovascular prevention in diabetes requires not only pharmacological management but also public health strategies aimed at improving access to screening, prevention programs, and long-term disease management.

Cultural background and ethnicity also influence cardiometabolic risk. In Europe, South Asian populations experience a disproportionately burden of T2D and CV risk factors compared with White European populations [73]. More broadly, contemporary observational studies show persistent ethnic differences in CV risk that are only partly explained by socioeconomic, lifestyle, and clinical factors [74]. Consistently, recent systematic reviews indicate that the prevalence and pattern of diabetes-related complications differ between ethnic minority groups and host populations, underscoring the need for targeted and culturally tailored prevention strategies [75]. Collectively, these data support the implementation of context-specific lifestyle interventions—including culturally adapted recommendations, community-based prevention programs, and targeted education strategies—combined with equitable access to CV risk screening and evidence-based treatment [76].

### 6.2. Overview of Pharmacological Interventions

The management of T2D has been profoundly transformed by the introduction of glucose-lowering agents that provide CV and renal protection beyond glycemic control. SGLT2i reduce hyperglycemia by promoting urinary glucose excretion and consistently lower the risk of hospitalizations, kidney disease progression, and major CV events (MACE) across a broad spectrum of patients, irrespective of baseline CVD status [77]. GLP-1 RAs enhance glucose-dependent insulin secretion and exert pleiotropic effects including appetite suppression, weight reduction, improved insulin sensitivity, and attenuation of systemic inflammation, translating into significant CV risk reduction [78,79,80,81]. More recently, dual incretin agonists, targeting both the glucose-dependent insulinotropic polypeptide (GIP) and GLP-1 receptors, have emerged as a highly effective therapeutic strategy for obesity and T2D. Tirzepatide demonstrated reductions in HbA1c and body weight across the SURPASS program. In SURPASS-2, tirzepatide was superior to semaglutide in improving glycemic control and body weight, with mean weight loss exceeding 10–15% depending on dose [82]. The SURPASS-CVOT demonstrated non-inferiority of tirzepatide compared with dulaglutide for major CV outcomes in patients with T2D and established atherosclerotic CVDs (hazard ratio 0.92, 95.3% CI 0.83–1.01; *p* = 0.003 for non-inferiority), confirming CV safety while maintaining superior metabolic efficacy [83]. Despite their clinical benefits, the widespread implementation of incretin-based therapies may be limited by cost, access disparities, and long-term adherence. Moreover, for newer dual- and multi-agonists, long-term cardiovascular outcome data remain limited, and real-world effectiveness across diverse populations requires further evaluation. Overall, both SGLT2i and incretin-based therapies—including GLP-1 RAs, dual (GLP-1/GIP), and emerging triple (GLP-1/GIP/glucagon) receptor agonists—favorable modulate multiple CV risk factors such as hypertension, dyslipidemia, renal dysfunction, hepatic fibrosis, and systemic inflammation, thereby contributing to comprehensive CV risk reduction [84,85,86,87]. Given the established association between severe hypoglycemia and adverse CV outcomes, preferential use of agents with a low hypoglycemic risk profile is of particular importance [88].

Dyslipidemia represents a key modifiable risk factor in diabetes-associated CVD. With regard to lipid-lowering therapies, statins are the cornerstone of lipid-lowering therapy, significantly reducing low-density lipoprotein cholesterol (LDL-C) levels and atherosclerotic CV events across risk categories [60]. In patients who fail to achieve lipid targets, adjunctive therapies such as ezetimibe, bempedoic acid or proprotein convertase subtilisin/kexin type 9 (PCSK9) inhibitors are recommended to further reduce residual risk [89]. Recent ESC focused updates emphasize early initiation of intensive lipid-lowering therapy during acute coronary syndromes, the measurement of Lp(a) for refined risk stratification, and targeted treatment of hypertriglyceridemia to address residual CV risk [32].

Optimal blood pressure control is fundamental for CV risk reduction in diabetes. Current guidelines recommend ACE inhibitors or ARBs as first-line therapy due to their CV and renal protective effects. When additional blood pressure lowering is required, calcium channel blockers or thiazide-like diuretics may be added [34]. Mineralocorticoid receptor antagonists (MRA) provide further benefit, particularly in patients with resistant hypertension or heart failure, including HFpEF [90].

Regarding antithrombotic therapy, in patients with diabetes and established atherosclerotic CVD, low-dose aspirin is recommended for secondary prevention. In selected high-risk individuals, intensified antiplatelet strategies, including dual antiplatelet therapy, may be considered after careful bleeding risk assessment [91]. In patients with atrial fibrillation or high thromboembolic risk, non-vitamin K oral anticoagulants (NOACs) offer superior safety compared with vitamin K antagonists (VKAs) while effectively reducing stroke and systemic embolism. In primary prevention, evidence indicates that aspirin provides only modest reductions in MACE in patients with T2D without prior CVD, offset by increased bleeding risk and no mortality benefit, underscoring the importance of careful patient selection and shared decision-making [92].

### 6.3. Integrated Care Models

A multidisciplinary and patient-centered approach is particularly relevant in individuals with diabetes because cardiovascular complications rarely occur in isolation and often emerge as overlapping cardiometabolic phenotypes requiring coordinated therapeutic prioritization. Integrated care frameworks involving doctors of internal medicine, endocrinologists, cardiologists, nephrologists, and primary care physicians facilitate early risk stratification, coordinated treatment strategies, and longitudinal follow-up, thereby reducing fragmentation of care and improving cardiometabolic outcomes [93,94]. Recent perspectives suggest that integrated care in diabetes should not be viewed merely as collaboration among different specialties, but rather as a structured clinical ecosystem in which patient data, digital monitoring tools, and coordinated decision pathways converge to guide risk stratification and therapeutic prioritization [62]. Within this framework, the integration of electronic medical records, digital monitoring technologies and clinical decision-support systems may enable a more dynamic model of cardiometabolic care, shifting the focus from episodic consultations to continuous risk assessment and adaptive treatment strategies [95]. In this context, a key objective of integrated care models is to allow clinicians to focus on high-value care activities—such as the interpretation of metabolic data, patient phenotyping, and therapeutic decision-making—while low-value or repetitive tasks can increasingly be supported by digital tools, allied health professionals, or automated data analysis systems. Moreover, patient education, structured lifestyle counselling, and the use of digital health and telemedicine-based interventions are tools useful to enhance self-management capabilities and adherence to therapeutic strategies in people with diabetes. Emerging data indicate that telemedicine and mobile health (mHealth) approaches are associated with improved glycemic control and better engagement in chronic disease management, supporting their integration into multidisciplinary care models [96].

## 7. Precision Medicine Approaches to Cardiovascular Risk in Diabetes

Ongoing research continues to explore novel therapeutic targets and precision and personalized medicine approaches, including biomarker-guided therapy selection and phenotype-specific intervention strategies aimed at reducing the burden of diabetes-related CV disease. Examples include the use of circulating biomarkers such as natriuretic peptides, inflammatory markers, or albuminuria to refine cardiometabolic risk stratification and guide therapeutic decisions [17,22,97]. In addition, advanced imaging techniques, including echocardiography-based cardiac phenotyping, coronary artery calcium scoring, and assessment of ectopic fat distribution, may help identify specific cardiometabolic phenotypes and subclinical organ damage [14,64]. Advances in genomics and data-driven approaches, including artificial intelligence and machine learning, are increasingly being explored to refine CV risk stratification and support more individualized prevention strategies in patients with diabetes [98]. In parallel, the development of next-generation cardioprotective antidiabetic agents, including other dual-, triple- and poly-agonists targeting multiple metabolic pathways, represents a promising avenue for enhancing metabolic control and addressing residual CV risk. These agents integrate effects on glucose metabolism, body weight, lipid profile, and inflammation, although their long-term CV efficacy requires confirmation in dedicated outcome trials.

Within this evolving framework, different clinical phenotypes can be recognized in patients with T2D, each characterized by distinct CV risk profiles and therapeutic priorities. In particular, patients may present with a predominance of atherosclerotic cardiovascular disease (ASCVD), heart failure-related phenotypes, chronic kidney disease with albuminuria, or obesity and metabolic liver disease-driven cardiometabolic risk. Identifying these patterns may help clinicians prioritize specific therapeutic strategies and monitoring approaches according to the dominant disease mechanism.

Taken together, these advances support a shift toward phenotype-driven, individualized management of CV risk in T2D, integrating clinical evaluation, diagnostic findings, and targeted therapeutic priorities, as summarized in Table 2.

## 8. Conclusions

Diabetes and cardiovascular disease are closely intertwined within a complex, multisystem framework that extends far beyond glycemic dysregulation alone. Multiple converging mechanisms, including insulin resistance, inflammation, lipotoxicity, and microvascular dysfunction, contribute to a broad spectrum of cardiovascular manifestations, explaining the persistent residual risk observed in individuals with diabetes. and highlighting the limitations of glucose-centered strategies alone. Over the past decade, the management paradigm has shifted from a glucose-centered approach to a comprehensive cardiometabolic strategy that integrates early cardiovascular risk stratification with multifactorial interventions. Glucose-lowering agents with proven cardiovascular and renal benefits, particularly SGLT2 inhibitors and GLP-1 receptor agonists, now represent key components of this approach, alongside management of lipids, blood pressure, weight, and other modifiable risk factors. Dual incretin agonists further expand therapeutic options by achieving substantial metabolic improvements, with cardiovascular safety established in high-risk populations. Optimal care extends beyond pharmacotherapy. Integrated, multidisciplinary, and patient-centered care models, combined with lifestyle interventions, education, and equitable access to preventive strategies, are essential to address the global and heterogeneous burden of diabetes-related cardiovascular disease. Emerging advances in precision medicine, digital health, and next-generation multi-agonist therapies may further refine individualized prevention and treatment in the coming years. In conclusion, addressing cardiovascular disease in diabetes requires a holistic, integrated approach that combines early risk detection, multifactorial intervention, and coordinated care across specialties. Such a strategy is essential to improve long-term cardiovascular outcomes and reshape the prognosis of individuals living with diabetes.

## Figures and Tables

**Table 1:** Major cardiovascular risk factors in type 2 diabetes and their estimated impact on cardiovascular risk.

Risk Factor	Mechanism/Clinical Relevance	Estimated Impact on CV Risk
**Hyperglycemia** [3]	Promotes oxidative stress, inflammation, and endothelial dysfunction	Each 1% increase in HbA1c associated with ~14–18% higher risk of myocardial infarction
**Duration of diabetes** [3,4]	Longer exposure leads to cumulative vascular injury and progressive atherosclerosis	Progressive increase in CV risk with longer disease duration
**Hypertension** [24,34]	Mechanical vascular stress and endothelial injury accelerate atherosclerosis	~2× higher risk of stroke and major CV events
**Dyslipidemia** [32,60]	Major driver of atherosclerotic plaque formation	Each 1 mmol/L reduction in LDL-C reduces major CV events by ~20–25%
**Smoking** [7]	Promotes oxidative stress, endothelial dysfunction, and thrombosis	Approximately 2–3× higher risk of cardiovascular events
**Chronic kidney disease** [2,61]	Marker of systemic endothelial dysfunction and microvascular injury	Associated with ~2–4× higher risk of CV mortality
**Atrial fibrillation** [44]	Increases risk of cardioembolic stroke	Diabetes associated with ~40% higher risk of AF
**Peripheral arterial disease** [19,20]	Marker of diffuse atherosclerosis and systemic vascular disease	ABI < 0.9 associated with markedly increased risk of major CV events

HbA1c: glycated hemoglobin; CV: cardiovascular; LDL-C: low-density lipoprotein cholesterol; AF: atrial fibrillation; ABI: ankle–brachial index.

**Table 2:** Phenotype-driven management of cardiovascular risk in type 2 diabetes.

Predominant Phenotype	Main Targets	Priority Cardiometabolic Therapies	Key Investigations	Follow-Up Focus
**ASCVD-predominant**	Prevention of recurrent ischemic events; stabilization of atherosclerosis.	High-intensity statin ± ezetimibe/PCSK9 inhibitor; antiplatelet therapy for secondary prevention; GLP-1 RAs and/or SGLT2i with proven CV benefit; ACE inhibitor/ARB if indicated.	ECG; lipid profile; renal function; assessment for PAD (ABI if symptomatic); targeted ischemia testing when clinically indicated.	Surveillance for recurrent ischemia; optimization of lipid and blood pressure control; adherence and bleeding risk assessment.
**HF/HFpEF-predominant**	Reduction of heart-failure hospitalizations; symptom control; preservation of functional capacity.	SGLT2i as foundational therapy; ACE inhibitor/ARB or ARNI as indicated; mineralocorticoid receptor antagonist; diuretics for congestion; GLP-1RAs mainly for metabolic control.	ECG; natriuretic peptides (BNP/NT-proBNP); echocardiography (systolic and diastolic function); renal function and electrolytes.	Symptom trajectory; volume status; renal function; prevention of decompensation.
**CKD-predominant**	Slowing kidney disease progression; reduction of CV risk.	SGLT2i; ACE inhibitor or ARB; non-steroidal mineralocorticoid receptor antagonist (finerenone) in persistent albuminuria; statin therapy.	eGFR; urinary albumin-to-creatinine ratio; serum potassium; blood pressure.	Kidney function decline; albuminuria response; hyperkaliemia risk; CV event prevention.
**Obesity/MASLD-predominant**	Weight reduction; improvement of metabolic and hepatic parameters; global CV risk reduction.	GLP-1 RAs or dual incretin agonist; lifestyle-based weight loss interventions; SGLT2i as complementary therapy; statin if indicated.	BMI and waist circumference; liver enzymes; non-invasive fibrosis assessment when indicated; cardiometabolic risk profiling.	Weight trajectory; metabolic control; liver disease progression; long-term CV risk.

ASCVD: atherosclerotic cardiovascular disease, PCSK9: proprotein convertase subtilisin/kexin type 9, GLP-1 RAs: glucagon-like peptide-1 receptor agonists, SGLT2i: sodium–glucose cotransporter 2 inhibitors, CV: cardiovascular, ACE: angiotensin-converting enzyme, ARB: angiotensin II receptor blocker, ECG: electrocardiogram, PAD: peripheral arterial disease, ABI: Ankle-Brachial Index, HFpEF: heart failure with preserved ejection fraction, ARNI: angiotensin receptor–neprilysin inhibitor, NT-proBNP: N-terminal pro–B-type natriuretic peptide, CKD: chronic kidney disease, eGFR: estimated glomerular filtration rate, MASLD: Metabolic dysfunction-associated steatotic liver disease, BMI: body mass index.

## Data Availability

Data sharing is not applicable to this article as no new data were created or analyzed in this study.

## Abbreviations

T2D (Type 2 Diabetes); CVD (Cardiovascular Disease); ASCVD (Atherosclerotic Cardiovascular Disease); CAD (Coronary Artery Disease); HF (Heart Failure); HFpEF (Heart Failure with Preserved Ejection Fraction); AF (Atrial Fibrillation); PAD (Peripheral Artery Disease); CKD (Chronic Kidney Disease); MASLD (Metabolic-Dysfunction-Associated Steatotic Liver Disease); HbA1c (Glycated Hemoglobin); ABI (Ankle–Brachial Index); CFR (Coronary Flow Reserve); ECG (Electrocardiogram); BNP (B-type Natriuretic Peptide); NT-proBNP (N-terminal pro–B-type Natriuretic Peptide); LDL-C (Low-Density Lipoprotein Cholesterol); HDL-C (High-Density Lipoprotein Cholesterol); Lp(a) (Lipoprotein(a)); eGFR (Estimated Glomerular Filtration Rate); RAAS (Renin–Angiotensin–Aldosterone System).
